# Acral Melanoma in Skin of Color: Current Insights and Future Directions: A Narrative Review

**DOI:** 10.3390/cancers17030468

**Published:** 2025-01-30

**Authors:** Emily R. Nadelmann, Ajay K. Singh, Matteo Abbruzzese, Oluwaseyi O. Adeuyan, Divya B. Kenchappa, Katherine Kovrizhkin, Michelle Lightman, Avishai Samouha, Kevin L. Tao, Jaewon Yun, Tian R. Zhu, Beth N. McLellan, Yvonne M. Saenger

**Affiliations:** 1 Department of Dermatology, Albert Einstein College of Medicine, Bronx, NY 10461, USA; enadelma@montefiore.org (E.R.N.); tzhu1@montefiore.org (T.R.Z.); bmclella@montefiore.org (B.N.M.); 2 Department of Oncology, Albert Einstein College of Medicine, Bronx, NY 10461, USA; ajay.singh2@einsteinmed.edu (A.K.S.); divya.kenchappa@einsteinmed.edu (D.B.K.); 3 Department of Medicine, Albert Einstein College of Medicine, Bronx, NY 10461, USA; matteo.abbruzzese@einsteinmed.edu (M.A.); katherine.kovrizhkin@einsteinmed.edu (K.K.); michelle.lightman@einsteinmed.edu (M.L.); avishai.samouha@einsteinmed.edu (A.S.); kevin.tao@einsteinmed.edu (K.L.T.); jaewon.yun@einsteinmed.edu (J.Y.); 4 Department of Medicine, Columbia University Vagelos College of Physicians and Surgeons, New York, NY 10032, USA; ooa2134@cumc.columbia.edu

**Keywords:** acral lentiginous melanoma (ALM), genetic mutation, BRAF, RAS, NF1, TWM, KIT, minorities, black, Hispanic

## Abstract

*Introduction*: Acral lentiginous melanoma (ALM), a rare subtype, accounts for 2–3% of melanoma cases, primarily affecting the palms, soles, and nail beds and disproportionately affects people of color. This review focuses on clinical insights into ALM and its management, with a focus on race and ethnicity. *Methods*: A comprehensive literature search was conducted in public databases using the search term “acral melanoma,” and studies focusing on epidemiology, clinical presentation, and treatment outcomes of ALM in various racial and ethnic groups were reviewed. *Results*: Significant disparities in ALM outcomes exist across racial and ethnic groups, with African, Hispanic, and Asian individuals presenting with thicker, more advanced tumors at diagnosis. These populations encounter unique challenges, including limited access to dermatologic care, under-recognition of melanoma presentation in darker skin types, and socioeconomic barriers leading to delayed diagnosis and treatment. Surgical management may require specialized approaches, such as partial amputation for subungual melanomas. Additionally, there is uncertainty regarding the tumor immune microenvironment (TME) in ALM, with some studies suggesting that it might be less favorable, resulting in a lower response to immunotherapy. *Conclusions*: ALM affects diverse populations, and the impact of ethnic and racial origin on ALM biology is largely unknown. Addressing disparities in ALM outcomes among racial and ethnic groups is critical for improving patient care. Increased awareness of melanoma risk in individuals with darker skin can significantly impact early detection and treatment. Future research should focus on the genetic and biological factors contributing to morbidity and mortality in ALM patients.

## Simple Summary

Acral lentiginous melanoma (ALM) presents notable challenges due to its distinct biological characteristics and disparities in outcomes among racial and ethnic groups. This review highlights genetic features and clinical features of ALM as well as the need for targeted interventions to address racial and ethnic disparities in ALM outcomes. Public health campaigns to increase awareness of melanoma risk in individuals with darker skin, along with improved access to dermatological screening, are critical to reducing these disparities. Furthermore, research into potential genetic and biological factors contributing to the differing incidence and prognosis among racial groups remains an important area for future study. These findings underscore the necessity of more inclusive research and tailored treatment approaches to improve ALM outcomes across all populations.

## 1. Introduction

**Figure 1:**
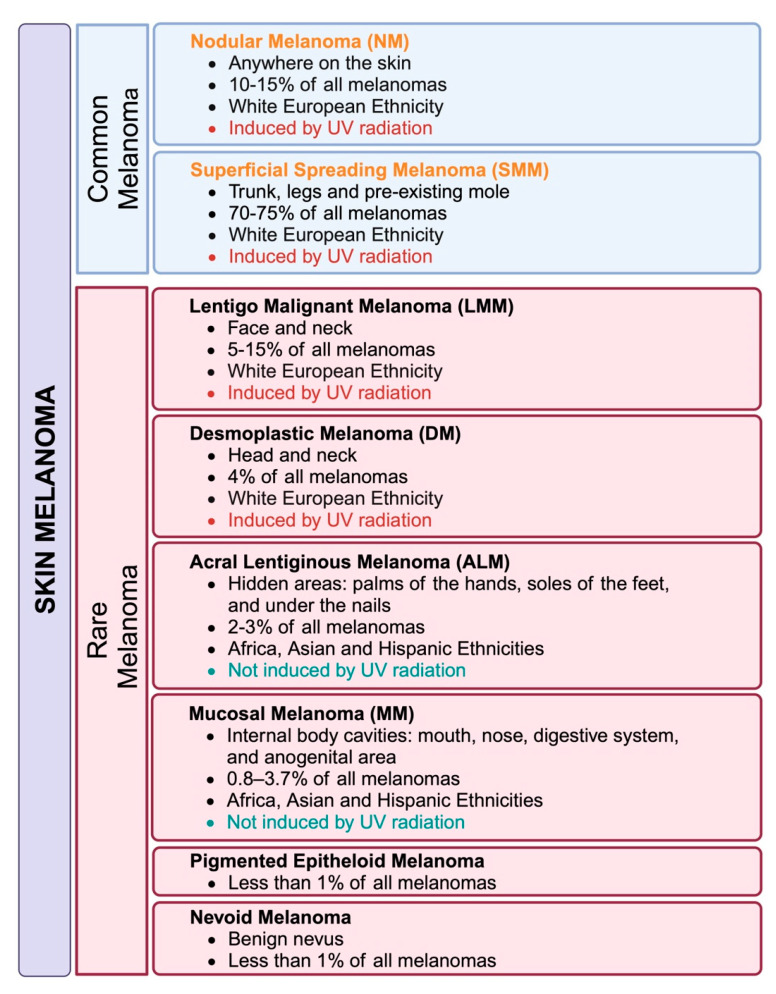
Systemic classification of acral lentiginous melanoma (ALM).

Acral melanoma (AM), also known as acral lentiginous melanoma (ALM), is a rare subtype of melanoma that predominantly occurs on the palms, soles, and nail beds (Figure 1). ALM is an uncommon subtype of melanoma, comprising only 2–3% of total melanoma diagnoses overall in Western countries but accounting for 55–65% of cases among patients of color [1]. ALM differs from cutaneous malignant melanoma because it is not induced by exposure to ultraviolet radiation (UVR), and studies have shown that ALM has a lower tumor mutation burden (TMB) and decreased immune infiltration [2,3]. The incidence of ALM is similar across populations of Asian, Hispanic, African, and European origin. However, people of color have a higher proportion of ALM than do European Whites [4].

Analysis of surveillance, epidemiology, and end result (SEER) databases has shown that underrepresented ethnic groups in the US experience lower melanoma-specific survival rates compared to patients of European White ancestry despite the introduction of advanced therapies [5]. This may be due to socioeconomic disparities as well as distinct genetic alterations or molecular profiles that contribute to unequal melanoma care and outcomes [6]. Higher socioeconomic status is associated with early diagnosis, surgical treatment, availability of immunotherapy, and improved survival among melanoma patients [7]. Notably, ALM patients with pigmented skin frequently present with thicker tumors and have a higher frequency of ulcerated tumors at diagnosis, all features that are associated with delayed survival outcomes and predicted poor prognosis. In contrast, non-Hispanic Whites (NHWs) demonstrate the lowest mean tumor thickness and ulceration rate in ALM [4]. Thicker primary ALMs at diagnosis affects prognosis as therapy is initiated later in the disease course [8]. The specific impact of the ALM subtype on prognosis remains largely undefined, although it has been hypothesized to contribute to poorer outcomes in patients with skin of color [9]. In this review, clinical features of ALM are reviewed with a focus on race and ethnicity.

## 2. Genetic Features and Predisposition to ALM

Defined genetic mutations are associated with melanomagenesis, resulting in uncontrolled cell proliferation. ALM has a genetic profile different from other melanomas because, classically, the palms and soles of the feet are not exposed to UV radiation (Figure 1). Therefore, other factors, such as genetic predisposition, environmental exposures, and stress in the affected skin, may contribute to oncogenesis. The genetic predisposition to melanoma can be attributed to specific genetic lesions as well as to genetic familial syndromes. While a higher proportion of Hispanic, Asian, and Black melanoma patients are affected by ALM, this may not reflect a higher risk profile in pigmented populations, but rather be attributed to the fact that pigment protects against classic UV induced cutaneous melanoma. Around 50–58% of melanomas in East Asian countries (Japan, China, Taiwan, and Korea) are reported to be ALM, and the incidence is similar in Latin American countries (Mexico and Peru). In South Africa, 65% of melanomas are ALM [1,10]. A study conducted in the US between 1986 and 2005 showed that 36% of the Black population with melanoma was diagnosed ALM (1,413 invasive cases of ALM).

Mutations common in melanoma, including BRAF, NRAS, and NF1 activate downstream signaling via the mitogen-activated protein kinase (MAPK) pathway and are more commonly found in non-ALM. ALM has also been reported to have a low tumor mutational burden with a median of 2.1 mutations per megabase [11] and can, in fact, be distinguished based on genetics from other subtypes with 89% accuracy [12]. ALM is characterized by a high level of DNA rearrangement, with whole genome duplication, complex genomic rearrangements and the presence of aneuploidy as well as a high rate of whole genome duplication [11].

Rates of classic melanoma-associated mutations in ALM are generally lower than those observed in the non-ALM prevalent in White European ethnicities. Depending on the population studied, approximately 50% of ALMs do carry mutations in BRAF, RAS, or NF1, but the absence of these mutants categorizes the ALM (around 50%) as a distinct group known as the triple-wild-type melanoma (TWM) [13] (Figure 1) TWM-associated mutational genes include KIT, CTNB1, CCND1, KDR, BCL2, MDM2, AKT3, IDH1, GNAQ, GNAS, CDKN2A, CDK4, MITF, RB1, TP53, APC, ERBB2, ERBB3, ABCB5, NUAK2, and TERT [13]. About 10–35% of ALM are BRAF mutated, ∼15–30% are NRAS mutated at exons 2 and 3, ∼11–23% are NF1 mutated, ∼45–58% carry mutations in TWT. KIT mutations range from ∼3–43% (Table 1) [14,15].

Studies of ALM genetics have yielded differing results depending on the geographic location of the patients studied. An Australian study found that a UV mutation signature is in fact detectable in a subset of ALM, which also has a higher mutation burden [11]. A study in Sweden revealed that 17% of ALM was associated with BRAF mutations and 15% with NRAS and KIT mutations [25]. A Korean population study showed 19.4% (7/36 cases) of ALM patients carried a BRAF gene mutation [26]. Furthermore, Taiwanese ALM patients were found to harbor the following mutations: BRAF (30%; V600E and V600L), NRAS (10%; G13R and G12D), MEK1 (5%; C121S), and PTEN (7.5%; Y315stop). This study also reported that oncogenic events may differ among melanomas in Asian carried patients geographically, such as between Taiwanese and Japanese patients [27]. In 2019, a study conducted in the United States by Yeh et al. included 122 cases of ALM. They found mutation in BRAF (21.3%; V600E, V600K, K601E, and G469S), NRAS (32.0%; Q61, G12, and G13), and KIT (11.5%; W557R, V559D, T574Q, L576P, K642E, D816V, D820Y, D820G, and N822K). ALM mortality in this population was correlated to tumor thickness and stage with no relation to race and ethnicity. Interestingly, the BRAF (V600E) mutation was found to be more prevalent in patients with European ancestry, although this was statistically non-significant (*p* = 0.09) [28]. This suggests that the genetic lesions in ALM may vary across populations and possibly be impacted by race, ethnicity, and specific toxic exposures.

KIT mutations in the central European cohort with white ethnicity are relatively common in ALM, reported at 43.04% (34 of 79 cases) [29]. Another study from the USA of 28 ALM patients similarly found a mutation rate of 36% as compared with 28% of patients with cutaneous melanoma [30]. Dia et al. reported that 43.6% (17/39 cases) of ALM in Chinese patients carried a KIT mutation [31]. A retrospective study (2005–2008) conducted at The University of Texas MD Anderson Cancer Center documented a 27% (46/173 cases) KIT mutation rate in primary ALM and a 14% rate (24/173 cases) in ALM metastasis [32]. In a cohort of Asians with ALM, the frequency of KIT mutations found was 16.7% [1]. However, studies specifically examining the impact of ethnicity on KIT mutation rates in ALM are limited. There is no direct evidence showing a specific melanoma syndrome is linked with the ALM subtype, although this may be due to the relatively small numbers of cases studied [33,34].

## 3. Clinical Presentation

**Figure 2:**
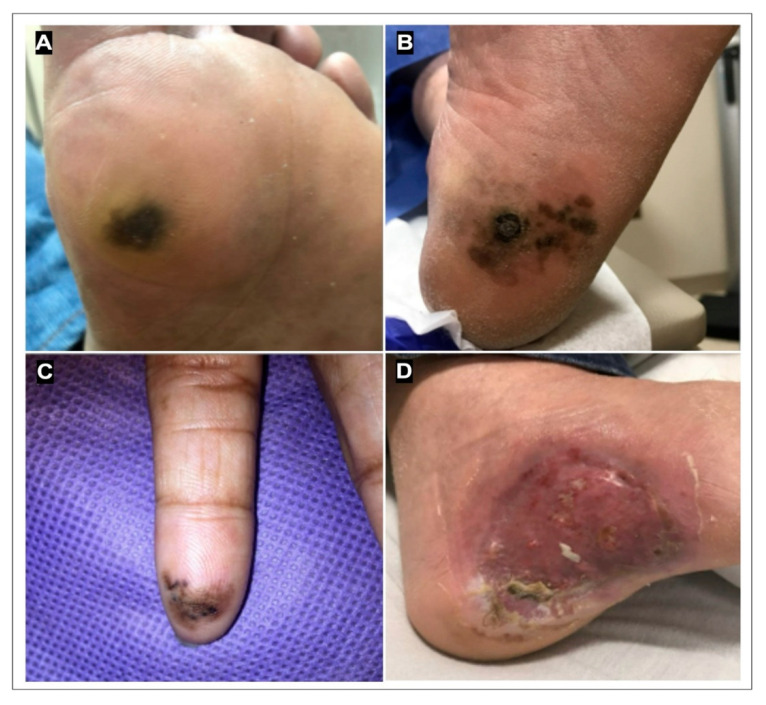
Acral lentiginous melanoma (ALM) in situ. (**A**) Symmetrical brown pigmented patch on the ball of the foot. (**B**) Irregular brown and black pigmented ulcer on the heel of the foot. (**C**) Irregular brown pigmented patch on the volar surface of the figure. (**D**) Ulcerated, amelanotic lesion on the arch of the foot.

Cutaneous malignant melanoma is commonly found on sun-exposed areas, characterized by the standard ABCDE (asymmetry, border irregularity, color variegation, diameter larger than 6 mm, evolution or timing of lesion’s growth) criteria [35]. However, ALM is not associated with UV exposure [36]. It is uniquely located on non-sun exposed areas with less pigmentation, including the palms, soles of feet, and nails [17]. On the palms and soles, ALM lesions often display symmetry, with homogenous dark brown or black pigmentation (Figure 2) [17]. They can also be amelanotic, characterized by a pink-red color. In advanced stages, ALM can present as a large, protruding nodule (Figure 2) [17]. Lesions underneath nail beds are normally long pigmented streaks, extending to the nail fold, that can cause splitting of the nail (Figure 2) [37]. The uncommon presentation of ALM makes the standard ABCDE criteria less useful for characterizing and diagnosing ALM, and the acronym CUBED (colored, bleeding lesions, uncertain diagnosis, enlarged, deteriorate with delayed healing) has been proposed to better assess lesions located on the feet, hands, and nail beds [38]. ALM has been commonly reported following trauma.

The unique clinical presentation of ALM poses diagnostic challenges in individuals with skin of color [39]. This oftentimes leads to a delay in diagnosis and results in advanced disease progression and poor prognosis [38]. ALM’s subtle pigment changes, unique anatomic location, [38] and atypical morphology, make lesions difficult to discern in individuals of skin of color, and may lead to diagnostic delay or misdiagnosis as benign conditions [40]. Delays in diagnosis can be further exacerbated by a lack of awareness of ALM among patients and providers [41]. As previously noted, melanoma is rare in individuals with skin of color, and potentially malignant lesions may be overlooked due to a lack of vigilance. These considerations must be considered when examining and educating individuals with skin of color to prevent delays in diagnosis. As suggested by the literature, dermoscopy may be important in the skin of color, as it can more accurately differentiate early, malignant lesions of ALM from benign lesions [38,42]. Ultimately, the combined effects of ALM’s unique clinical presentation, diagnostic challenges, combined with a lack of patient education and awareness, lead to much later diagnosis, poorer prognosis, and higher rates of recurrence [42].

## 4. Management

As with other cutaneous melanomas, surgical wide local excision remains the first-line treatment for localized ALM, even in the presence of the Hutchinson’s sign. ALM lesions, especially those with residual pigmentation, may require wider margins given that ALM is frequently under-staged and that margin guidelines were developed for non-ALM lesions [43]. In the setting of positive margins, reflectance confocal microscopy may aid in precise resection of the residual melanoma [44]. Additionally, the exact surgical approach must be tailored to the specific location of ALM. Namely, subungual or distal digit melanoma may require partial amputation or phalanx bony resection to maintain a functional digit [45]. However, in proximal digit and/or web-space-based invasive melanomas, wide excision with preservation of underlying tendinous and bony structure can often be achieved followed by reconstruction with a skin graft [45].

Sentinel lymph node biopsy (SLNB) is recommended for patients with ALM < 0.8 mm with ulceration or >0.8–1.0 mm, with or without ulceration based on current NCCN guidelines [46]. There is controversy on the prognostic role of SLNB and the need for complete regional lymph node dissection [47]. In patients with localized regional spread, adjuvant radiation therapy may be considered for select patients. As ALM is rarer and often diagnosed at a later stage, there is limited evidence on the efficacy of specific systemic therapies for ALM [22]. Additionally, as there is a lack of dominant MAPK-activating mutations in ALM that can be targeted, targeted therapies and immunotherapies have been shown to be less efficacious in ALM [22]. While systemic therapies have been thoroughly researched in the adjuvant and neoadjuvant setting for cutaneous melanoma, there are limited specific data on their role in ALM [23].

Targeted therapies, such as BRAF and MEK inhibitors, can potentially offer promising treatment options for ALM, despite the relatively low frequency of BRAF mutations (~5–30%) in ALM patients [48,49]. The efficacy of BRAF/MEK inhibitor therapy for ALM, particularly with the BRAF V600E mutation has been explored in various studies, Jiang et al. demonstrated that treatment with the BRAF inhibitor vemurafenib in combination with the MEK inhibitor cobimetinib resulted in significant tumor responses and improved progression-free survival in patients with ALM and BRAF V600E mutations [50]. The phase 3 COMBI-v trial further confirmed that the combination of BRAF and MEK inhibitors significantly improved progression-free survival and response rates compared to BRAF monotherapy in ALM patients with BRAF mutations [51]. Additionally, Long et al. reported that BRAF/MEK combination therapy provided durable responses in patients with metastatic ALM, highlighting the potential of this targeted treatment strategy for advanced disease [52].

Immunotherapy with anti-programmed cell death 1 (anti-PD-1) therapy alone or in combination with anti-cytotoxic T lymphocyte antigen-4 (CTLA-4) antibody ipilimumab is currently established as first line for patients with metastatic melanoma. For Stage IIB and IIC cutaneous melanoma, single-agent immunotherapy is preferred [22]. The KEYNOTE-716 trial examined patients with completely resected stage IIB or IIC cutaneous melanoma treated with adjuvant pembrolizumab. However, this study did not report on histologic subtype analysis. Similarly, the CheckMate 76K trial enrolled patients with completely resected stage IIB or IIC cutaneous melanoma with adjuvant nivolumab or placebo. Those treated with nivolumab had a 58% lower risk of recurrence or death compared to placebo. Of note, ALM made up only 5.4% (*n* = 43) of the patient population [53].

In patients with stage III-IV cutaneous melanoma, the use of adjuvant pembrolizumab and adjuvant nivolumab has been supported by several trials. The EORTC 1325/KEYNOTE-054 Phase III trial found a significantly improved recurrence-free survival with adjuvant pembrolizumab compared to placebo for resected high-risk stage III melanoma [54]. Similarly, the Checkmate 238 Phase III trial compared adjuvant therapy with either nivolumab or ipilimumab for patients with resected stage IIIB-C or IV cutaneous melanoma and found a significantly improved recurrence-free survival (RFS) with nivolumab [55]. Nivolumab also showed superiority to ipilimumab in a 5-year follow-up study of the Checkmate 238 trial, in which patients treated with nivolumab had a 50% RFS while those treated with ipilimumab only had 39% [55]. However, none of these studies reported histological subtype analysis to ascertain the proportion of ALM in the study population.

While several other clinical trials, including CheckMate 066, 067 and KEYNOTE-006, have established a survival benefit of treatment with immune checkpoint inhibitors, there is limited information available regarding the benefit for each subtype as well as the benefit of adjuvant versus neoadjuvant therapy in these patients [56,57,58].

Recent trials have demonstrated the potential benefits of neoadjuvant immunotherapy over adjuvant therapy in advanced cutaneous melanoma, and while these trials included patients with ALM, insufficient data exist to determine benefit in this subtype. The OpaCIN and OpACIN-neo trials demonstrated that neoadjuvant immune checkpoint inhibitors can induce robust immune responses and improve event-free survival compared to adjuvant therapy alone. However, these studies did not specify the representation of ALM within their patient cohorts [59]. SWOG S1801 similarly showed superior event-free survival, with neoadjuvant pembrolizumab followed by surgery and adjuvant pembrolizumab, compared to adjuvant therapy alone. This phase II trial included nine ALM participants, including four participants receiving neoadjuvant therapy and five receiving adjuvant pembrolizumab. Unfortunately, two of the nine patients assigned to adjuvant therapy only experienced fatal outcomes [23,60].

Most recently, the Phase III NADINA trial revealed a significant 68% reduction in the risk of recurrence of death with neoadjuvant nivolumab plus ipilimumab in resectable stage III melanoma as compared with adjuvant nivolumab. Although ALM was included in the cohort of 423 patients, the exact number of ALM cases within this cohort remains unclear [61]. The PRADO trial further investigated the potential of personalized response-driven approaches following neoadjuvant ipilimumab and nivolumab in high-risk stage III melanoma. This trial demonstrated that patients who achieved a major pathological response could forgo completion lymph node dissection and adjuvant therapy, potentially reducing treatment-related morbidity while maintaining favorable outcomes (Clinical Trial ID #NCT02977052) [62]. While ALM patients were included, detailed data on the exact number were not provided. Although specific data on ALM patients in these trials are limited, the inclusion of diverse melanoma subtypes underscores the potential applicability of neoadjuvant strategies for ALM, particularly in Caucasian patients, the predominant group enrolled in these studies.

Despite encouraging findings, ALM is believed to exhibit a lower responsiveness to immunotherapy compared to cutaneous melanoma. This is primarily attributed to its unique tumor microenvironment, which is characterized by a low mutational burden and limited neoantigen presentation. Unlike cutaneous melanomas, which are typically driven by UV-induced mutations, ALM tumors are associated with genetic alterations that are less antigenic, rendering them more difficult for the immune system to recognize [63]. Additionally, ALM tumors tend to harbor fewer tumor-infiltrating lymphocytes, critical for the efficacy of immune checkpoint inhibitors such as anti-PD-1 and anti-CTLA-4 [64]. The fact that ALM often arises in non-sun-exposed areas limits the influence of UV-induced immune pathways, likely reducing immunotherapy efficacy [65].

Beyond this, there may be other factors limiting the efficacy of immunotherapy aside from the subtype of melanoma itself. These include delayed detection of ALM, potential genetic differences influenced by ancestry, and unique interactions between genetics and the immune system that may affect immune sensitivity [1]. Environmental factors, such as UV exposure, and mechanical contributors, including trauma, could also play a role in its distinct biology [1]. However, these hypotheses remain underexplored.

Few studies have aimed to elucidate the impact of race or ethnicity on the efficacy of immunotherapy for ALM. It is hypothetically possible that UV signatures are more prevalent in ALM in fair-skinned Caucasians. Nakamura et al., retrospectively examined 193 advanced-stage Japanese ALM patients treated with anti-PD-1 antibodies and, surprisingly, found that anti-PD-1 antibodies with lower response rates were observed in patients with nail apparatus melanoma than those with palm and sole melanoma. Bhave et al., also sought to assess the efficacy of checkpoint inhibitors in 325 patients with unresectable stage III/IV ALM [66]. They found that the objective response rate (ORR) to the anti-PD-1/ipilimumab combination was significantly higher than that of single-agent anti-PD-1 (43% vs. 26%) and notably higher than in the Japanese study. However, the observed increased ORR with combination immunotherapy did not translate into improved overall survival in this retrospective analysis. Notably, 76% of the patients in the cohort were European, and the analysis revealed no significant association between ORR and race (non-White vs. White).

Intralesional talimogene laherparepvc (T-VEC), an oncolytic virus immunotherapy, has been studied as monotherapy and in combination with systemic therapy. It was approved by the Food and Drug Administration (FDA) after the landmark OPTiM trial in the United States, which found that T-VEC was well-tolerated among 436 patients and resulted in a higher durable response rate (DRR) and longer median overall survival (OS) when compared to granulocyte-macrophage colony-stimulating factor (GM-CSF) [67]. Oncolytic viral therapy could be useful for melanomas of the lower extremity suitable for injection. However, this study did not account for histologic subtypes.

As ALM often recurs in the distal extremities, regional therapy approaches are also being investigated for ALM. However, there are still limited data regarding efficacy. Similarly, isolated limb infusion (ILI) is also being studied for patients with ALM. One large study in China investigated ILI in 150 patients with cutaneous melanoma with in-transit metastases, with ALM representing 79% of the total study population [68]. In this trial, a complete response (CR) occurred in 6% of the total study population and a partial response (PR) in 35%. Those with CR or PR to ILI had better in-field progression-free survival (PFS) and OS [68].

In summary, given the limited and conflicting findings regarding immunotherapy efficacy in ALM subtype of melanoma, there is a need for more studies involving diverse populations, with a specific focus on race and ethnicity. Furthermore, in the setting of the unique mutational landscape of ALM and relatively low frequency of BRAF mutations, the efficacy of BRAF and MEK inhibitors has been more limited in patients with ALM [14], although there have been multiple reports of benefit of c-KIT inhibitors in KIT mutated ALM [69].

## 5. Prognosis

ALM, although relatively uncommon, represents one of the most lethal forms of melanoma. Similar to other melanoma subtypes, factors such as Breslow depth and sentinel lymph node involvement significantly influence prognosis. However, ALM exhibits a worse prognosis due to additional factors, notably its disproportionate impact on patients of varying skin tones. ALM constitutes approximately 2–3% of melanoma diagnoses but is the most prevalent melanoma subtype among individuals with darker skin tones, including Hispanic, African American, and Asian populations [70]. Studies examining post-diagnosis survival rates of ALM across different populations reveal persistent survival disparities, even when controlling disease severity. This suggests socioeconomic factors beyond the biology of ALM subtype are contributing to the worse prognosis observed in certain groups [4,13].

Several factors may account for the poorer prognosis of ALM in patients with skin of color. Socioeconomic status plays a significant role, influencing healthcare access and leading to infrequent medical consultations, which are associated with advanced-stage presentations and poorer outcomes. Additionally, delayed presentation to healthcare providers often stems from a lack of awareness regarding risk factors and clinical manifestations of ALM. Diagnostic delays also contribute to a worse prognosis. Physicians may exhibit a lower index of suspicion for ALM in patients with certain skin colors, leading to later diagnoses [4,13]. Ethnicity may impact survival in ALM patients. In the United States, survival rates for patients with ALM vary by race and were reported to be 82.6–69.4% in non-Hispainc Whites, 77.2–71.5% in Blacks, 72.8–57.3% in Hispanic Whites, and 70.2–54.1% in Asian Islanders [4].

Emerging prognostic markers and predictive models are poised to revolutionize the management of melanoma, particularly ALM, by leveraging advanced genetic and molecular insights. Recent studies have highlighted the prognostic and predictive value of genetic markers such as mutations in BRAF, NRAS, and KIT, which are also found in ALM. Genetic profiling of tumors enables the identification of high-risk mutations, thereby informing prognosis and guiding treatment decisions [71]. Moreover, molecular markers, including gene expression profiles of microRNAs and long non-coding RNAs, have been implicated in melanoma progression [14], while biomarkers like PD-L1 expression, CD8 infiltration, and interferon related gene expression can predict response to immunotherapy [11]. The integration of genetic and molecular markers into clinical practice facilitates personalized treatment strategies, utilizing targeted therapies (e.g., BRAF inhibitors) and immunotherapies for suitable patients. The development of predictive models that amalgamate clinical, genetic, and molecular data can forecast disease behavior and treatment responses with enhanced accuracy [71].

Addressing disparities in healthcare access, increasing awareness of risk factors and clinical presentations, and improving follow-up care can mitigate the poorer prognosis of ALM in patients with skin of color. Educating both patients and physicians about the unique presentation of ALM in various skin tones can facilitate earlier diagnosis and improve outcomes. Additionally, patient-specific treatments, including targeted immunotherapy tailored to individual mutation statuses and logistical considerations regarding follow-up care, can enhance treatment efficacy and prognosis for these patients [23,70,71,72,73].

## 6. Variation in Incidence and Outcomes of ALM Across Different Racial and Ethnic Groups

Examining ALM across different races and ethnicities is crucial due to the significant variations in presentation, response to treatment, and outcomes. There is a paucity of literature addressing the differences in these trends. A study by Holman et al., utilizing data from the Centers for Disease Control (CDC) and Prevention’s National Program of Cancer Registries (NPCR) and the National Cancer Institute’s Surveillance, Epidemiology, and End Results Program (SEER), analyzed ALM incidence rates from 2010 to 2019 across various racial and ethnic groups [16]. They found that, while ALM is a high proportion of melanomas in patients of color, ALM incidence overall is lower among non-Hispanic Black, non-Hispanic Asian/Pacific Islander, and Hispanic Black/American Indian/Alaska Native populations, with higher rates observed in Hispanic White individuals compared to non-Hispanic Whites. The study underscores the need to recognize ALM’s rarity while noting the higher prevalence of other melanoma types in certain groups [16].

A recent study by Ansbro et al. analyzed data from 22 SEER registries to assess trends in the incidence and mortality of ALM across different racial and ethnic groups from 2000 to 2020 [74]. They found that the incidence of ALM increased annually by 2.48%, primarily due to rising rates among Hispanics and non-Hispanic Whites, while rates remained stable for non-Hispanic Blacks and non-Hispanic Asians/Pacific Islanders. Additionally, Hispanics, non-Hispanic Asians/Pacific Islanders, and non-Hispanic Blacks had significantly higher ALM-specific mortality compared to non-Hispanic Whites, highlighting the need for targeted strategies to address these health disparities [74]. The additive effect of socioeconomic status on survival in ethnic minorities with ALM was highlighted by a Brazilian study showing correlation of income with survival [10].

## 7. Strategies to Improve Outcomes in ALM

As previously discussed, multiple systemic and socioeconomic factors impact the diagnosis and management of ALM in patients with skin of color. Several strategies, particularly in patient education and medical instruction, have been developed to address these factors and ultimately bridge the health disparity gap. Several studies have recognized that most public-facing educational materials, such as social media platforms, are primarily tailored to non-Hispanic White individuals, thus inadvertently contributing to late detection of melanoma in people with skin of color [75,76,77]. In a study by Grewel et al. investigating social media posts of 10 well-established organizations in skin cancer prevention, the authors found that 100% of the total images used to depict skin cancer examples across the sites were in Fitzpatrick type I or II individuals, which represent the lightest skin types [77]. Moreover, of these images, only 3.2% (2 out of 62) depicted skin cancer in typical locations for ALM or mucosal melanoma, including palmoplantar, nail, and mucosal areas. These findings contribute to the misinformation among individuals with skin of color that skin cancer occurs only in sun-exposed areas of people with light skin [41,76,78]. Similarly, many existing educational materials for patients and physicians have historically highlighted the ABCDE rule as a tool for standard melanoma detection, which has limited utility in the acral subtype. Instead, the “CUBED” rule has been found to be a more sensitive indicator [79].

In addition to the content of patient education materials, the delivery of medical information is also implicated in ALM management. Video instruction has been found to be an effective means of delivering information on skin cancer recognition and protection when compared to traditional text materials [80,81,82]. A subsequent study by Alvarado and Feng also found that online materials, such as VisualDx, included greater representation of individuals with darker skin when compared to standard print materials, which may consequently have positive impact on the receipt and implementation of skin cancer knowledge in communities of color [76,83].

In a recent study by Jaklitsch et al., pamphlets on ALM designed with dual input from dermatologists and community health leaders were distributed to multiple centers in the Pittsburgh area, representing a community-engaged educational initiative for ALM awareness [84]. Given the high response rate across diverse participants, in addition to the increased awareness of ALM and positive attitudes towards skin cancer surveillance post-intervention, the authors found that print materials with community input may serve as a method for addressing racially disparate outcomes in ALM. Additional strategies that have been proposed include promoting the use of the “CUBED” system in a broad range of health professionals (e.g., podiatrists) and industries (e.g., nail salons), recruiting more underrepresented groups in clinical trials, deriving models of ALM from a spectrum of skin tones for preclinical research and emphasizing transparency regarding the inclusion of patients with skin of color in medical decision-making and scientific initiatives to ensure applicability to the general population [85].

## 8. Conclusions

ALM poses significant challenges, particularly in individuals with skin of color, where delayed diagnosis and poorer outcomes remain prevalent due to a combination of biological, socioeconomic, and healthcare-related factors [4]. Combating these disparities requires a comprehensive approach, including heightened awareness among both healthcare providers and patients, improved early detection strategies, and enhanced access to care. Future research should aim to elucidate the genetic and molecular drivers of ALM in diverse populations and to develop therapies that target these specific mechanisms. By promoting healthcare literacy and access to care, and prioritizing equity in both research and treatment, we can work towards personalizing care for ALM patients and narrowing the outcome gap for this aggressive melanoma subtype.

## Figures and Tables

**Table 1:** Summary for Acral Lentiginous Melanoma (ALM).

Features	Acral Lentiginous Melanoma (ALM)	References
Demographics	More common in darker skin types (Fitzpatrick skin types IV–VI)	[16]
	35–70% in Black, Hispanic, and Asian populations	
	Mean age: 60 years	
Clinical Features	Arises on acral sites (palms, soles, subungual)	[17,18]
	Presents as irregularly pigmented macules or patches, melanonychia in subungual lesions	
	Preceded by trauma	
Genetics	Low BRAF (B-Raf proto-oncogene, serine/threonine kinase) mutations (15–30%)	[1,19]
	High KIT (v-kit Hardy-Zuckerman 4 feline sarcoma viral oncogene homolog) mutations (3–36%)	
	Frequent DNA rearrangement (whole genome duplication, aneuploidy, and complex rearrangements)	
	Lower tumor mutational burden (TMB)	
Histopathology	Lentiginous growth with atypical melanocytes	[20]
	Vertical growth phase in advanced stages, often with necrosis and ulceration	
Management	Excisional biopsy (1–2 mm margins)	[21]
	Wide local excision (WLE) for definitive treatment	
	Sentinel lymph node biopsy (SLNB) for lesions ≥T1b or >0.8 mm	
Treatment	Localized: Wide local excision (WLE)	[22,23,24]
	Adjuvant: Anti-programmed cell death protein 1 (anti-PD1)	
	Advanced: Immune checkpoint inhibitors (anti-PD1/CTLA-4, cytotoxic T-lymphocyte-associated protein 4), KIT-targeted therapy (Imatinib)	
Prognosis	5-year survival: ~50–60% for localized; lower for advanced	[6]
	KIT mutations, tumor thickness, ulceration, and lymph node involvement are poor prognostic factors	
