# Correction: Nadelmann et al. Acral Melanoma in Skin of Color: Current Insights and Future Directions: A Narrative Review. *Cancers* 2025, *17*, 468

**DOI:** 10.3390/cancers17152458

**Published:** 2025-07-25

**Authors:** Emily R. Nadelmann, Ajay K. Singh, Matteo Abbruzzese, Oluwaseyi O. Adeuyan, Divya B. Kenchappa, Katherine Kovrizhkin, Michelle Lightman, Avishai Samouha, Kevin L. Tao, Jaewon Yun, Tian R. Zhu, Beth N. McLellan, Yvonne M. Saenger

**Affiliations:** 1 Department of Dermatology, Albert Einstein College of Medicine, Bronx, NY 10461, USA; enadelma@montefiore.org (E.R.N.); tzhu1@montefiore.org (T.R.Z.); bmclella@montefiore.org (B.N.M.); 2 Department of Oncology, Albert Einstein College of Medicine, Bronx, NY 10461, USA; ajay.singh2@einsteinmed.edu (A.K.S.); divya.kenchappa@einsteinmed.edu (D.B.K.); 3 Department of Medicine, Albert Einstein College of Medicine, Bronx, NY 10461, USA; matteo.abbruzzese@einsteinmed.edu (M.A.); katherine.kovrizhkin@einsteinmed.edu (K.K.); michelle.lightman@einsteinmed.edu (M.L.); avishai.samouha@einsteinmed.edu (A.S.); kevin.tao@einsteinmed.edu (K.L.T.); jaewon.yun@einsteinmed.edu (J.Y.); 4 Department of Medicine, Columbia University Vagelos College of Physicians and Surgeons, New York, NY 10032, USA; ooa2134@cumc.columbia.edu

## Additional Affiliations

In the published publication [1], one affiliation was missing. The corrected list now includes a total of four affiliations. The correct Affiliations 1, 3, and 4 are provided below:

1 Department of Dermatology, Albert Einstein College of Medicine, Bronx, NY 10461, USA; enadelma@montefiore.org (E.R.N.); tzhu1@montefiore.org (T.R.Z.); bmclella@montefiore.org (B.N.M.)
3 Department of Medicine, Albert Einstein College of Medicine, Bronx, NY 10461, USA; matteo.abbruzzese@einsteinmed.edu (M.A.); katherine.kovrizhkin@einsteinmed.edu (K.K.); michelle.lightman@einsteinmed.edu (M.L.); avishai.samouha@einsteinmed.edu (A.S.); kevin.tao@einsteinmed.edu (K.L.T.); jaewon.yun@einsteinmed.edu (J.Y.)
4 Department of Medicine, Columbia University Vagelos College of Physicians and Surgeons, New York, NY 10032, USA; ooa2134@cumc.columbia.edu

## Text Correction

There was an error in the original publication [1] regarding the heading sequence in the Abstract. A correction has been made to the **Abstract** section as follows:

**Abstract**: *Introduction*: Acral lentiginous melanoma (ALM), a rare subtype, accounts for 2–3% of melanoma cases, primarily affecting the palms, soles, and nail beds and disproportionately affects people of color. This review focuses on clinical insights into ALM and its management, with a focus on race and ethnicity. *Methods*: A comprehensive literature search was conducted in public databases using the search term “acral melanoma,” and studies focusing on epidemiology, clinical presentation, and treatment outcomes of ALM in various racial and ethnic groups were reviewed. *Results*: Significant disparities in ALM outcomes exist across racial and ethnic groups, with African, Hispanic, and Asian individuals presenting with thicker, more advanced tumors at diagnosis. These populations encounter unique challenges, including limited access to dermatologic care, under-recognition of melanoma presentation in darker skin types, and socioeconomic barriers leading to delayed diagnosis and treatment. Surgical management may require specialized approaches, such as partial amputation for subungual melanomas. Additionally, there is uncertainty regarding the tumor immune microenvironment (TME) in ALM, with some studies suggesting that it might be less favorable, resulting in a lower response to immunotherapy. *Conclusions*: ALM affects diverse populations, and the impact of ethnic and racial origin on ALM biology is largely unknown. Addressing disparities in ALM outcomes among racial and ethnic groups is critical for improving patient care. Increased awareness of melanoma risk in individuals with darker skin can significantly impact early detection and treatment. Future research should focus on the genetic and biological factors contributing to morbidity and mortality in ALM patients.

There was an error in the original publication [1] due to a grammatical mistake. A correction has been made in the **Introduction**, **Paragraph 2**:

Analysis of surveillance, epidemiology, and end result (SEER) databases has shown that underrepresented ethnic groups in the US experience lower melanoma-specific survival rates compared to patients of European White ancestry despite the introduction of advanced therapies [5]. This may be due to socioeconomic disparities as well as distinct genetic alterations or molecular profiles that contribute to unequal melanoma care and outcomes [6]. Higher socioeconomic status is associated with early diagnosis, surgical treatment, availability of immunotherapy, and improved survival among melanoma patients [7]. Notably, ALM patients with pigmented skin frequently present with thicker tumors and have a higher frequency of ulcerated tumors at diagnosis, all features that are associated with delayed survival outcomes and predicted poor prognosis. In contrast, non-Hispanic Whites (NHWs) demonstrate the lowest mean tumor thickness and ulceration rate in ALM [4]. Thicker primary ALMs at diagnosis affects prognosis as therapy is initiated later in the disease course [8]. The specific impact of the ALM subtype on prognosis remains largely undefined, although it has been hypothesized to contribute to poorer outcomes in patients with skin of color [9]. In this review, clinical features of ALM are reviewed with a focus on race and ethnicity.

There was an error in the original publication [1] due to a grammatical mistake. A correction has been made to the section **Genetic Features and Predisposition to ALM**, **Paragraph 2**:

Mutations common in melanoma, including BRAF, NRAS, and NF1 activate downstream signaling via the mitogen-activated protein kinase (MAPK) pathway and are more commonly found in non-ALM. ALM has also been reported to have a low tumor mutational burden with a median of 2.1 mutations per megabase [11] and can, in fact, be distinguished based on genetics from other subtypes with 89% accuracy [12]. ALM is characterized by a high level of DNA rearrangement, with whole genome duplication, complex genomic rearrangements and the presence of aneuploidy as well as a high rate of whole genome duplication [11].

There was an error in the original publication [1] due to a grammatical mistake. A correction has been made to the section **Genetic Features and Predisposition to ALM**, **Paragraph 4**:

Studies of ALM genetics have yielded differing results depending on the geographic location of the patients studied. An Australian study found that a UV mutation signature is in fact detectable in a subset of ALM, which also has a higher mutation burden [11]. A study in Sweden revealed that 17% of ALM was associated with BRAF mutations and 15% with NRAS and KIT mutations [25]. A Korean population study showed 19.4% (7/36 cases) of ALM patients carried a BRAF gene mutation [26]. Furthermore, Taiwanese ALM patients were found to harbor the following mutations: BRAF (30%; V600E and V600L), NRAS (10%; G13R and G12D), MEK1 (5%; C121S), and PTEN (7.5%; Y315stop). This study also reported that oncogenic events may differ among melanomas in Asian carried patients geographically, such as between Taiwanese and Japanese patients [27]. In 2019, a study conducted in the United States by Yeh et al. included 122 cases of ALM. They found mutation in BRAF (21.3%; V600E, V600K, K601E, and G469S), NRAS (32.0%; Q61, G12, and G13), and KIT (11.5%; W557R, V559D, T574Q, L576P, K642E, D816V, D820Y, D820G, and N822K). ALM mortality in this population was correlated to tumor thickness and stage with no relation to race and ethnicity. Interestingly, the BRAF (V600E) mutation was found to be more prevalent in patients with European ancestry, although this was statistically non-significant (*p* = 0.09) [28]. This suggests that the genetic lesions in ALM may vary across populations and possibly be impacted by race, ethnicity, and specific toxic exposures. 

There was an error in the original publication [1] due to a grammatical mistake. A correction has been made to the section **Genetic Features and Predisposition to ALM**, **Paragraph 5**:

KIT mutations in the central European cohort with white ethnicity are relatively common in ALM, reported at 43.04% (34 of 79 cases) [29]. Another study from the USA of 28 ALM patients similarly found a mutation rate of 36% as compared with 28% of patients with cutaneous melanoma [30]. Dia et al. reported that 43.6% (17/39 cases) of ALM in Chinese patients carried a KIT mutation [31]. A retrospective study (2005–2008) conducted at The University of Texas MD Anderson Cancer Center documented a 27% (46/173 cases) KIT mutation rate in primary ALM and a 14% rate (24/173 cases) in ALM metastasis [32]. In a cohort of Asians with ALM, the frequency of KIT mutations found was 16.7% [1]. However, studies specifically examining the impact of ethnicity on KIT mutation rates in ALM are limited. There is no direct evidence showing a specific melanoma syndrome is linked with the ALM subtype, although this may be due to the relatively small numbers of cases studied [33,34].

There was an error in the original publication [1] due to a grammatical mistake. A correction has been made to the section **Clinical Presentation**, **Paragraph 1**:

Cutaneous malignant melanoma is commonly found on sun-exposed areas, characterized by the standard ABCDE (asymmetry, border irregularity, color variegation, diameter larger than 6 mm, evolution or timing of lesion’s growth) criteria [35]. However, ALM is not associated with UV exposure [36]. It is uniquely located on non-sun exposed areas with less pigmentation, including the palms, soles of feet, and nails [17]. On the palms and soles, ALM lesions often display symmetry, with homogenous dark brown or black pigmentation (Figure 2) [17]. They can also be amelanotic, characterized by a pink-red color. In advanced stages, ALM can present as a large, protruding nodule (Figure 2) [17]. Lesions underneath nail beds are normally long pigmented streaks, extending to the nail fold, that can cause splitting of the nail (Figure 2) [37]. The uncommon presentation of ALM makes the standard ABCDE criteria less useful for characterizing and diagnosing ALM, and the acronym CUBED (colored, bleeding lesions, uncertain diagnosis, enlarged, deteriorate with delayed healing) has been proposed to better assess lesions located on the feet, hands, and nail beds [38]. ALM has been commonly reported following trauma. 

There was an error in the original publication [1] due to a grammatical mistake. A correction has been made to the section **Clinical Presentation**, **Paragraph 2**:

The unique clinical presentation of ALM poses diagnostic challenges in individuals with skin of color [39]. This oftentimes leads to a delay in diagnosis and results in advanced disease progression and poor prognosis [38]. ALM’s subtle pigment changes, unique anatomic location, [38] and atypical morphology, make lesions difficult to discern in individuals of skin of color, and may lead to diagnostic delay or misdiagnosis as benign conditions [40]. Delays in diagnosis can be further exacerbated by a lack of awareness of ALM among patients and providers [41]. As previously noted, melanoma is rare in individuals with skin of color, and potentially malignant lesions may be overlooked due to a lack of vigilance. These considerations must be considered when examining and educating individuals with skin of color to prevent delays in diagnosis. As suggested by the literature, dermoscopy may be important in the skin of color, as it can more accurately differentiate early, malignant lesions of ALM from benign lesions [38,42]. Ultimately, the combined effects of ALM’s unique clinical presentation, diagnostic challenges, combined with a lack of patient education and awareness, lead to much later diagnosis, poorer prognosis, and higher rates of recurrence [42].

There was an error in the original publication [1] due to a grammatical mistake. A correction has been made to the section **Management**, **Paragraph 1**:

As with other cutaneous melanomas, surgical wide local excision remains the first-line treatment for localized ALM, even in the presence of the Hutchinson’s sign. ALM lesions, especially those with residual pigmentation, may require wider margins given that ALM is frequently under-staged and that margin guidelines were developed for non-ALM lesions [43]. In the setting of positive margins, reflectance confocal microscopy may aid in precise resection of the residual melanoma [44]. Additionally, the exact surgical approach must be tailored to the specific location of ALM. Namely, subungual or distal digit melanoma may require partial amputation or phalanx bony resection to maintain a functional digit [45]. However, in proximal digit and/or web-space-based invasive melanomas, wide excision with preservation of underlying tendinous and bony structure can often be achieved followed by reconstruction with a skin graft [45].

There was an error in the original publication [1] due to a grammatical mistake. A correction has been made to the section **Management**, **Paragraph 4**:

Immunotherapy with anti-programmed cell death 1 (anti-PD-1) therapy alone or in combination with anti-cytotoxic T lymphocyte antigen-4 (CTLA-4) antibody ipilimumab is currently established as first line for patients with metastatic melanoma. For Stage IIB and IIC cutaneous melanoma, single-agent immunotherapy is preferred [22]. The KEYNOTE-716 trial examined patients with completely resected stage IIB or IIC cutaneous melanoma treated with adjuvant pembrolizumab. However, this study did not report on histologic subtype analysis. Similarly, the CheckMate 76K trial enrolled patients with completely resected stage IIB or IIC cutaneous melanoma with adjuvant nivolumab or placebo. Those treated with nivolumab had a 58% lower risk of recurrence or death compared to placebo. Of note, ALM made up only 5.4% (*n* = 43) of the patient population [53]. 

There was an error in the original publication [1] due to a grammatical mistake. A correction has been made to the section **Management**, **Paragraph 6**:

While several other clinical trials, including CheckMate 066, 067 and KEYNOTE-006, have established a survival benefit of treatment with immune checkpoint inhibitors, there is limited information available regarding the benefit for each subtype as well as the benefit of adjuvant versus neoadjuvant therapy in these patients [56–58].

There was an error in the original publication [1] due to a grammatical mistake. A correction has been made to the section **Management**, **Paragraph 7**:

Recent trials have demonstrated the potential benefits of neoadjuvant immunotherapy over adjuvant therapy in advanced cutaneous melanoma, and while these trials included patients with ALM, insufficient data exist to determine benefit in this subtype. The OpaCIN and OpACIN-neo trials demonstrated that neoadjuvant immune checkpoint inhibitors can induce robust immune responses and improve event-free survival compared to adjuvant therapy alone. However, these studies did not specify the representation of ALM within their patient cohorts [59]. SWOG S1801 similarly showed superior event-free survival, with neoadjuvant pembrolizumab followed by surgery and adjuvant pembrolizumab, compared to adjuvant therapy alone. This phase II trial included nine ALM participants, including four participants receiving neoadjuvant therapy and five receiving adjuvant pembrolizumab. Unfortunately, two of the nine patients assigned to adjuvant therapy only experienced fatal outcomes [23,60].

There was an error in the original publication [1] due to an incomplete sentence. A correction has been made to the section **Management**, **Paragraph 9**:

Despite encouraging findings, ALM is believed to exhibit a lower responsiveness to immunotherapy compared to cutaneous melanoma. This is primarily attributed to its unique tumor microenvironment, which is characterized by a low mutational burden and limited neoantigen presentation. Unlike cutaneous melanomas, which are typically driven by UV-induced mutations, ALM tumors are associated with genetic alterations that are less antigenic, rendering them more difficult for the immune system to recognize [63]. Additionally, ALM tumors tend to harbor fewer tumor-infiltrating lymphocytes, critical for the efficacy of immune checkpoint inhibitors such as anti-PD-1 and anti-CTLA-4 [64]. The fact that ALM often arises in non-sun-exposed areas limits the influence of UV-induced immune pathways, likely reducing immunotherapy efficacy [65]. 

There was an error in the original publication [1] due to an incomplete sentence. A correction has been made to the section **Management**, **Paragraph 11**:

Few studies have aimed to elucidate the impact of race or ethnicity on the efficacy of immunotherapy for ALM. It is hypothetically possible that UV signatures are more prevalent in ALM in fair-skinned Caucasians. Nakamura et al., retrospectively examined 193 advanced-stage Japanese ALM patients treated with anti-PD-1 antibodies and, surprisingly, found that anti-PD-1 antibodies with lower response rates were observed in patients with nail apparatus melanoma than those with palm and sole melanoma. Bhave et al., also sought to assess the efficacy of checkpoint inhibitors in 325 patients with unresectable stage III/IV ALM [66]. They found that the objective response rate (ORR) to the anti-PD-1/ipilimumab combination was significantly higher than that of single-agent anti-PD-1 (43% vs. 26%) and notably higher than in the Japanese study. However, the observed increased ORR with combination immunotherapy did not translate into improved overall survival in this retrospective analysis. Notably, 76% of the patients in the cohort were European, and the analysis revealed no significant association between ORR and race (non-White vs. White). 

There was an error in the original publication [1] due to an incomplete sentence. A correction has been made to the section **Management**, **Paragraph 14**:

In summary, given the limited and conflicting findings regarding immunotherapy efficacy in ALM subtype of melanoma, there is a need for more studies involving diverse populations, with a specific focus on race and ethnicity. Furthermore, in the setting of the unique mutational landscape of ALM and relatively low frequency of BRAF mutations, the efficacy of BRAF and MEK inhibitors has been more limited in patients with ALM [14], although there have been multiple reports of benefit of c-KIT inhibitors in KIT mutated ALM [69].

There was an error in the original publication [1] due to a grammatical mistake. A correction has been made to the section **Prognosis**, **Paragraph 1**:

ALM, although relatively uncommon, represents one of the most lethal forms of melanoma. Similar to other melanoma subtypes, factors such as Breslow depth and sentinel lymph node involvement significantly influence prognosis. However, ALM exhibits a worse prognosis due to additional factors, notably its disproportionate impact on patients of varying skin tones. ALM constitutes approximately 2–3% of melanoma diagnoses but is the most prevalent melanoma subtype among individuals with darker skin tones, including Hispanic, African American, and Asian populations [70]. Studies examining post-diagnosis survival rates of ALM across different populations reveal persistent survival disparities, even when controlling disease severity. This suggests socioeconomic factors beyond the biology of ALM subtype are contributing to the worse prognosis observed in certain groups [4,13].

There was an error in the original publication [1] due to a grammatical mistake. A correction has been made to the section **Prognosis**, **Paragraph 2**:

Several factors may account for the poorer prognosis of ALM in patients with skin of color. Socioeconomic status plays a significant role, influencing healthcare access and leading to infrequent medical consultations, which are associated with advanced-stage presentations and poorer outcomes. Additionally, delayed presentation to healthcare providers often stems from a lack of awareness regarding risk factors and clinical manifestations of ALM. Diagnostic delays also contribute to a worse prognosis. Physicians may exhibit a lower index of suspicion for ALM in patients with certain skin colors, leading to later diagnoses [4,13]. Ethnicity may impact survival in ALM patients. In the United States, survival rates for patients with ALM vary by race and were reported to be 82.6–69.4% in non-Hispainc Whites, 77.2–71.5% in Blacks, 72.8–57.3% in Hispanic Whites, and 70.2–54.1% in Asian Islanders [4].

There was an error in the original publication [1] due to a grammatical mistake. A correction has been made to the section **Variation in Incidence and Outcomes of ALM Across Different Racial and Ethnic Groups**, **Paragraphs 1 and 2**:

Examining ALM across different races and ethnicities is crucial due to the significant variations in presentation, response to treatment, and outcomes. There is a paucity of literature addressing the differences in these trends. A study by Holman et al., utilizing data from the Centers for Disease Control (CDC) and Prevention’s National Program of Cancer Registries (NPCR) and the National Cancer Institute’s Surveillance, Epidemiology, and End Results Program (SEER), analyzed ALM incidence rates from 2010 to 2019 across various racial and ethnic groups [16]. They found that, while ALM is a high proportion of melanomas in patients of color, ALM incidence overall is lower among non-Hispanic Black, non-Hispanic Asian/Pacific Islander, and Hispanic Black/American Indian/Alaska Native populations, with higher rates observed in Hispanic White individuals compared to non-Hispanic Whites. The study underscores the need to recognize ALM’s rarity while noting the higher prevalence of other melanoma types in certain groups [16].

A recent study by Ansbro et al. analyzed data from 22 SEER registries to assess trends in the incidence and mortality of ALM across different racial and ethnic groups from 2000 to 2020 [74]. They found that the incidence of ALM increased annually by 2.48%, primarily due to rising rates among Hispanics and non-Hispanic Whites, while rates remained stable for non-Hispanic Blacks and non-Hispanic Asians/Pacific Islanders. Additionally, Hispanics, non-Hispanic Asians/Pacific Islanders, and non-Hispanic Blacks had significantly higher ALM-specific mortality compared to non-Hispanic Whites, highlighting the need for targeted strategies to address these health disparities [74]. The additive effect of socioeconomic status on survival in ethnic minorities with ALM was highlighted by a Brazilian study showing correlation of income with survival [10].

The authors apologize for any inconvenience caused and state that the scientific conclusions are unaffected. This correction was approved by the Academic Editor. The original publication has also been updated.
